# Comprehensive Analysis of the Safety Profile of a Single-Stranded RNA Nano-Structure Adjuvant

**DOI:** 10.3390/pharmaceutics11090464

**Published:** 2019-09-07

**Authors:** Hyeong-Jun Park, Hae Li Ko, Dong-Hoon Won, Da-Bin Hwang, Yoo-Sub Shin, Hye-Won Kwak, Hye-Jung Kim, Jun-Won Yun, Jae-Hwan Nam

**Affiliations:** Department of Biotechnology, The Catholic University of Korea, Bucheon 14662, Korea (H.-J.P.) (H.L.K.) (D.-H.W.) (D.-B.H.) (Y.-S.S.) (H.-W.K.) (H.-J.K.)

**Keywords:** nano-structure adjuvant, vaccine, ssRNA, autoimmune disease

## Abstract

Adjuvants enhance the efficacy of vaccines by stimulating immune response-related gene expression and pathways. Although some adjuvants have been approved for commercial use in human vaccines (e.g., Alum, MF59, and AS03), they might elicit adverse side effects, such as autoimmune diseases. Recently, we developed a novel single-stranded RNA (ssRNA) nano-structure adjuvant, which can stimulate both Th1 and Th2 responses. In this study, we evaluated the safety and toxicological profiles of this ssRNA nano-structure adjuvant in vitro and in vivo. Mice were intramuscularly immunized with the ssRNA nano-structure adjuvant three times, once every 2 weeks. The results indicate no significant differences in hematological and serum biochemistry parameters between the ssRNA-treated groups and the control group. From a histopathological perspective, no evidence of tissue damage was found in any group. The levels of IgE and anti-nuclear antibodies, which are markers of autoimmune disease, were not different between the ssRNA-treated groups and the control group. The findings of this study suggest that the ssRNA nano-structure can be used as a safe adjuvant to increase vaccine efficacies.

## 1. Introduction

Current vaccines are based on various strategies used in an attempt to protect against pathogen infection [1,2,3]. Among them, formulating vaccines with an adjuvant typically increases vaccine efficiency. The adjuvant can recruit antigen-presenting cells (APCs), such as dendritic cells (DCs) and monocytes, or stabilize the vaccine. Recently, newer adjuvants have been developed to increase vaccine efficacy, especially for peptide-based vaccines [4,5,6,7]. Besides, some adjuvants are under development, such as the toll-like receptor (TLR) agonist, which also induces innate immune responses (a prerequisite for an effective vaccine) [4,8]. Since the discovery of adjuvants, peptide-based vaccines, subunit vaccines, and conjugated vaccines using protein antigens have been co-administered with adjuvants [6,7].

Despite ongoing efforts for more than 80 years, the number of currently approved adjuvants is minimal. In the United States, only five adjuvants have been approved: Alum, AS04, MF59, AS01, and CpG 1018 [6,9]. Among them, alum was approved as an adjuvant for use in humans and the squalene-based adjuvants MF59 and AS03 were approved in Europe and the USA for use with influenza vaccines [6,10,11,12]. However, alum mainly induces Th2-type immune responses and does not induce cell-mediated immune responses. MF59 is an excellent adjuvant for inducing antibody production against antigens, but poorly induces cell-mediated immune responses [9,10,11]. Moreover, the fact that these adjuvants are “licensed” does not mean that they are entirely safe. In case of the alum adjuvant, problems with autoimmune/inflammatory syndrome induced by adjuvants (ASIA syndrome) have been found, such as multiple sclerosis, systemic lupus erythematosus, and chronic fatigue syndrome [13]. Squalene-based adjuvants such as MF59 and AS03 are known to cause chronic arthritis in rats [14] or to induce lupus-related anti-nRNP/Sm/Su antibodies in mice [15]. Therefore, although the first consideration when developing an adjuvant is increased immunogenicity, the most crucial consideration is the safety of the adjuvant.

Immune cells in humans facilitate specific immune responses to infection with different pathogenic bacteria or viruses, but they have receptors to respond to conserved pathogen-associated molecular patterns associated with bacterial cell walls, lipopolysaccharide, proteins, or viral RNA/DNA [16]. These receptors are known as pattern-recognition receptors (PRRs). TLRs are typical PRRs, and TLR agonists highly activate innate immune responses. Thus, TLR agonists are being developed as vaccine adjuvants [16]. Most TLR ligands activate antigen presentation by macrophages, promote DC maturation, and upregulate MHC class 2, which in turn induces T cell responses [17,18]. Among several TLR agonists, double-stranded RNA (dsRNA) can induce innate immune responses via TLR-3 [19], making it is an excellent TLR-based adjuvant candidate. For example, poly I:C, a representative dsRNA, was tested as an adjuvant [20]. However, poly I:C induced systemically massive cytokine release (i.e., a cytokine storm), and the resulting excessive immune responses could finally lead to adverse effects [21,22]. Furthermore, poly ICLC, which is a poly I:C derivative, can cause severe pulmonary pathology [23]. Moreover, it has been shown that dsRNA can aggravate renal function [24]. In addition, since the immunological effect differed depending on the length of poly I:C, making standardization difficult, poly I:C is unsuitable for use as an adjuvant, even though it was capable of highly inducing immune responses [25].

Single-stranded RNA (ssRNA) is also known to serve as an agonist of TLR-7/8 and retinoic acid-inducible gene I (RIG-I), which can induce innate immune responses and increase immunogenicity [26,27]. Compared to dsRNA, ssRNA can induce appropriate immune responses and does not remain in the body for a long time [28,29], resulting in fewer intense adverse effects. Currently, ssRNA is being studied as an intrinsic adjuvant in tumor-vaccination studies [30,31,32]. Recently, the German biotech company CureVac AG has tested ssRNA nano-structure adjuvant formulations with disulfide-cross-linked cationic carrier peptides, CR_12_C [27,33] although ssRNA has not been clinically approved as an adjuvant. In addition, many researchers have been intensively studying the potential use of ssRNA as a vaccine adjuvant [34,35,36]. For instance, Lou et al. [34] have found that ssRNA nanocomplex adjuvant showed to be efficient at targeting lymph nodes and inducing Th1-type immunity. Edwards et al. [35] have identified the consistent adjuvant effects of a sequence-engineered mRNA vaccine, both in vitro human and in vivo mouse models. Heidenreich et al. [36] have demonstrated strong immunostimulatory capacities of a novel RNA-based adjuvant. Considering that ssRNA nano-structure adjuvants might be eventually applied for use with prophylactic vaccines, the safety issue is especially important.

Recently, we developed several ssRNA-expression platforms [37]. Among them, we selected RNA derived from the internal ribosome entry site (IRES) in the intergenic region (IGR) of cricket paralysis virus (CrPV) as an ssRNA nano-structure adjuvant, because it can express the desired gene in vivo and in vitro [37] and can elicit strong immune responses (including innate immune responses) through Th1-type immune responses [38,39]. Immunizing mice and non-human primates with CrPV IGR IRES-derived ssRNA and protein-based vaccine showed increased production of antibodies, including IgG1 (indicating Th2 responses) and IgG2 (indicating Th1 responses), and neutralizing antibodies [39,40]. Moreover, we previously found that the CrPV IGR IRES-derived ssRNA was clustered as a 25-nm sized nano-structure by atomic force microscopy [40]. Although it has been well documented that the ssRNA nano-structure can function as an immune stimulator, its safety has not been intensively studied. In this study, we verified the safety of a new ssRNA nano-structure adjuvant in a variety of ways. Herein, we present both the general toxicity profile and immunological-toxicity results in mice.

## 2. Materials and Methods

### 2.1. Adjuvant

The ssRNA nano-structure adjuvant was designed using IGR IRES and SV40 late-polyadenylation signal sequences [37]. Because it was designed as expression platform, the ssRNA nano-structure adjuvant has four restriction enzyme sequences between the untranslated regions that enable cloning into a multi-cloning site and insertion of various genes. In vitro transcription was performed using the EZ T7 High Yield In Vitro Transcription Kit (Enzynomics, Daejeon, Korea), according to the manufacturer’s instructions. SsRNA purification was performed using LiCl. The ssRNA concentrations were evaluated using a NanoDrop 2000 spectrophotometer (Thermo Fisher Scientific, MA, USA), and ssRNA integrity was analyzed by denaturing gel electrophoresis. Brief structure of ssRNA adjuvant is shown in Figure S1.

### 2.2. Cell Culture

HS68 cells, derived from human foreskin fibroblast were obtained from Korean Cell Line Bank (Seoul, Korea). HS68 cells were cultured in Dulbecco’s modified Eagle medium (HyClone™) with 10% fetal bovine serum (FBS, Corning, NY, USA) and 1% antibiotic–antimycotic (Anti–anti, Gibco, CA, USA). The cells were cultured in a humidified 37 °C incubator with 5% CO_2_.

### 2.3. Cell-Viability Assay

In vitro cell-viability testing was performed with a 3-(4,5-demerthylthiazol-2-yl)-2,5-diphenyltetrazolium bromide (MTT) assay. Hs68 cells were seeded in 24-well plates at densities of 2 × 10^5^ cells/well. After 24 h, the medium without 10% fetal bovine serum was replaced and then 20, or 200 μg of the ssRNA nano-structure adjuvant or poly I:C was added to each well. Cell viabilities were determined at different time points (24, 48, and 72 h) by performing MTT assays. The viability of cells in each well was measured in terms of the optical density at a wavelength of 570 nm, using a GloMax^®^ microplate reader (Promega, WI, USA).

### 2.4. Mice

Six-week-old BALB/c mice were purchased from Daehan Biolink Co. (Seoul, Korea). The mice were quarantined and acclimated for 1 week before initiating the study after evaluating their health status. Mice were housed at the Catholic University of Korea under specific-pathogen-free conditions with a 12-h light/dark cycle, a temperature of 23 ± 2 °C, and a relative humidity of 50% ± 10%. The animals were handled according to protocols approved by the Catholic University of Korea (10 June 2018). The animal facility at the Catholic University of Korea is fully accredited by the Korean Association for Laboratory Animals. All mice experimental procedures conducted in this study followed the guidelines of the Institutional Animal Care and Use Committee of the Catholic University of Korea (Approval No. CUK-IACUC-2018-026 and CUK-IACUC-2019-010).

### 2.5. Experimental Design for In Vivo Toxicity Study

**Figure 1:**
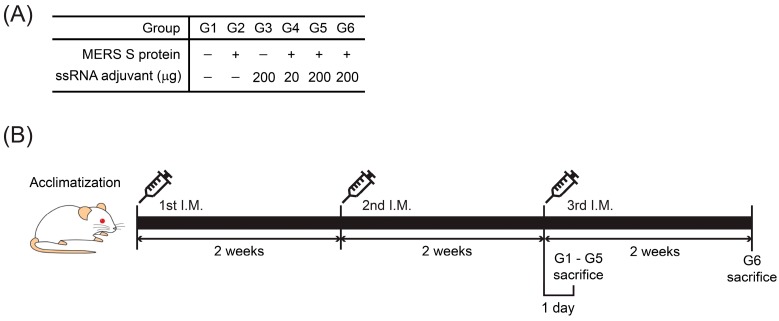
(**A**) Group design and (**B**) immunization schedule.

The experimental design for the toxicity assessments followed the guideline on the nonclinical evaluation of biopharmaceuticals from the Ministry of Food and Drug Safety [41]. To evaluate the toxicity of the ssRNA nano-structure adjuvant, mice (6-week-old) were randomly distributed into six groups (10 male and 10 female mice in each group). In this study, we used both sexes in compliance with the guidelines [41]. A protein-based vaccine candidate is Middle East Respiratory Syndrome Coronavirus (MERS-CoV) spike (S) protein, which was expressed in insect cells [39]. This MERS S protein was obtained from the International Vaccine Institute (Seoul, Korea). The six groups involved in this study were treated as follows: group 1 (G1), PBS-control group; group 2 (G2), 1 μg/mouse of MERS S protein; group 3 (G3), 200 μg/mouse of ssRNA nano-structure adjuvant; group 4 (G4), 1 μg/mouse of MERS S protein and 20 μg/mouse of ssRNA nano-structure adjuvant as the low-dose group; group 5 (G5), 1 μg/mouse of MERS S protein and 200 μg/mouse of ssRNA nano-structure adjuvant as the high-dose group; group 6 (G6), 1 μg/mouse of MERS S protein and 200 μg/mouse of ssRNA nano-structure adjuvant as the recovery group. Animals were intramuscularly immunized three times in a volume of 100 μL every 2 weeks, and blood was collected on the day before every immunization. Groups 1–5 were sacrificed 1 day after the last immunization. Also, we investigated group 6 recovery for another 2 weeks after the last immunization to assess long-lasting, delayed, or reversible toxic effects according to the guideline [41]. The protein and ssRNA doses used for immunization were the same or lower than those used in previous studies [39,42,43,44]. The group design and immunization schedules are shown in Figure 1.

In separate in vivo experiments conducted to analyze immunological responses, the same groups aforementioned were designed (five female mice in each group), but group 6 was immunized 2 weeks earlier than the other groups so that all mice were sacrificed on the same day. The mice in groups 1–5 were sacrificed 1 day after the last immunization, and those in group 6 were sacrificed 2 weeks after the last immunization. As a positive control for IgE analysis, eight female mice were administered 1% 2,4-dinitrochlorobenzene (DNCB) every other day for 3 weeks and sacrificed at 2 weeks after the last treatment. DNCB was dissolved in a 3:1 mixture of acetone and olive oil. The hair of all animals was removed the day before DNCB was applied to their backs, using a dose of 200 μL/mouse [45]. Animals were monitored for body weight and food intake every week. At the end of the study, the mice were anesthetized with isoflurane (2–5%). Blood samples were obtained from the abdominal aorta, and the mice were sacrificed. One mouse in group 2 died one day before the first immunization, for unknown reasons.

### 2.6. Hematological and Serum Biochemical Analysis In Vivo Toxicity Study

Blood samples were collected in tubes containing the anticoagulant, ethylenediaminetetraacetic acid. The following hematological parameters were measured with a ProCyte Dx hematology analyzer (IDEXX, ME, USA): total erythrocyte count (RBC), hemoglobin concentration (HGB), hematocrit (HCT), mean cell volume (MCV), mean cell hemoglobin (MCH), mean cell hemoglobin concentration (MCHC), platelet (PLT), total leucocyte count (WBC), and differential WBC (neutrophils, lymphocytes, monocytes, eosinophils, and basophils).

Serum samples were obtained by centrifuging blood samples at 2000× *g* for 30 min. The following biochemistry parameters were measured with a Mindray BS-220 chemical analyzer (Mindray, Shenzhen, China): alanine aminotransferase (ALT), aspartate aminotransferase (AST), total cholesterol (T-Chol), triglycerides (TG), glucose, high density lipoprotein cholesterol (HDL), low density lipoprotein cholesterol (LDL), blood urea nitrogen (BUN), creatinine, total protein (TP), albumin, and the albumin-to-globulin ratio (A/G).

### 2.7. Gross Findings, Organ Weights, and Histopathological Assessments In Vivo Toxicity Study

Immediately after the mice were sacrificed, several organs (liver, kidney, spleen, thymus, lung, heart, lymph node, muscle, brain, testis) were removed from the mice, examined macroscopically, and weighed. The organ weights relative to the terminal body weight were then calculated. The organs were fixed in 10% neutral buffered formalin for histopathological examination. The testis was fixed in Bouin’s solution. The fixed organs were processed for paraffin embedding. Paraffin sections were stained with hematoxylin and eosin (H + E). The microscopic features of the organs from mice in the control (G1) and high-dose groups (G5) were examined by an experienced pathologist, under a light microscope (Leica, Hamburg, Germany).

### 2.8. Mouse IgE Mouse Enzyme-Linked Immunosorbent Assays (ELISAs) In Vivo Toxicity Study

Serum samples were prepared by centrifugation (2000× *g*, 30 min) from the last collected blood sample of each group and stored at −80 °C. The levels of total serum IgE were measured using a Mouse Enzyme-Linked Immunosorbent Assay Kit (Bethyl Laboratory, Inc., TX, USA). Each step was performed according to the manufacturer’s instructions. IgE concentrations were determined based on the absorbance at 450 nm, as detected with a GloMax^®^ microplate reader (Promega, WI, USA).

### 2.9. Mouse Anti-Nuclear Antibody ELISAs In Vivo Toxicity Study

Serum samples were prepared by centrifugation (2000× *g*, 30 min) from the last collected blood sample of each group and stored at −80 °C. The levels of anti-nuclear antibodies were measured using a Mouse Anti-Nuclear Antibody IgG ELISA Kit (MyBioSource, Inc., CA, USA). Each step was performed according to the manufacturer’s instructions.

### 2.10. Cytokine Analysis In Vivo Toxicity Study

Serum samples were prepared by centrifugation (2000× *g*, 30 min) from the last collected blood sample of each group and stored at −80 °C. The concentrations of IL-1β, IL-6, IL-10, IL-12 p70, TNF-α, and MCP-1 were analyzed in each sample, using the Magnetic Luminex^®^ Screening Assay Kit (R&D Systems, Inc., MN, USA) in accordance with the manufacturer’s instructions.

### 2.11. Flow Cytometry Analysis In Vivo Toxicity Study

For surface staining, splenocytes and isolated immune cells from spleen, muscle, and lymph node tissues were stained for 15 min at room temperature with antibodies against the following proteins: CD4 (clone GK1.5, 863 eBioscience; clone H129.19, BioLegend, CA, USA), CD8 (clone 53-6.7, BD Biosciences; clone 53-6.7, Invitrogen, CA, USA), CD44 (clone IM7, Invitrogen), CD62L (clone MEL 14, BD Biosciences), CD11b (clone M1/70, Bio Legend), F4/80 (clone BM8, Invitrogen), CD86 (clone GL1, BD Biosciences), and CD11c (clone N48, eBioscience). Cells were fixed with 1% paraformaldehyde, analyzed using a FACS Canto II flow cytometer (BD Biosciences, NJ, USA), and the data were analyzed using FlowJo software (TreeStar, OR, USA).

### 2.12. Statistical Analysis

One-way analysis of variance was used to assess significant differences among the treatment groups. For each significant effect identified, Tukey’s honestly significant difference test and Bonferroni were used to compare multiple group means. In addition, all histomorphometric values are expressed as means ± standard deviations (SD). Multiple-comparison tests for the different treatment groups were conducted. If significant deviations from variance homogeneity were detected using the Levene test, then the non-parametric Kruskal–Wallis H-test was conducted. When a significant difference was detected by the Kruskal–Wallis H-test, a Mann–Whitney U-test was conducted as a post-hoc analysis. Statistical analyses were conducted using SPSS for Windows (release 14.0K, SPSS Inc., IL, USA). To assess significant differences between two groups, Student’s *t*-test was used. Differences were considered significant at *p* < 0.05.

## 3. Results

### 3.1. Cellular Toxicity of the ssRNA Nano-Structure Adjuvant In Vitro

**Figure 2:**
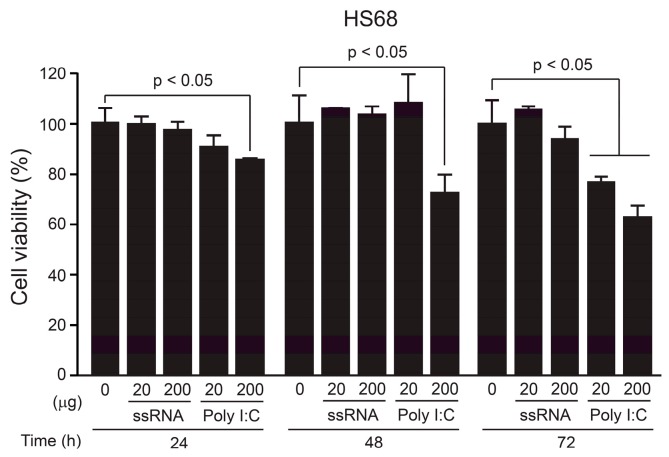
Dose-dependent cell viabilities of Hs68 cell line treated with the ssRNA nano-structure adjuvant, using MTT assays. Relative viabilities of Hs68 cells were compared to negative control (0 concentration of ssRNA nano-structure adjuvant) from 24 h to 72 h, based on the ssRNA concentration (20 and 200 μg). Poly I:C (20 and 200 μg) was used as a positive control. Unlike poly I:C, the ssRNA did not affect cell viability in Hs68 cells. The data were normalized to 100%. The data shown are expressed as the mean ± SD.

To investigate the toxicity of the ssRNA nano-structure adjuvant, we measured the viabilities of treated HepG2 and A549 cells, which are liver- and lung-derived tumor cell lines, respectively, [46,47] as well as Hs68, which is a human skin-derived normal cell line [48] in MTT assays. Previously, we injected mice with 20 μg of ssRNA nano-structure adjuvant to increase immune responses [39]. Based on this concentration, we treated the cells with various concentrations of the ssRNA nano-structure adjuvant (10–200 μg/well) for 24, 48, or 72 h. The ssRNA did not affect cell viability at any concentration tested in tumor cell lines (Figure S2A,B) and normal human cell lines (Figure 2). However, poly I:C as positive control showed some toxicity in the normal cell line (Figure 2). Furthermore, poly I:C induced higher pro-inflammatory cytokines than those of ssRNA nano-structure adjuvant in RAW 264.7 cells, which are a mouse macrophage cell line (Figure S3, detailed in supplementary methods), indicating poly I:C may stimulate a stronger inflammation response compared to that of the ssRNA nano-structure adjuvant.

### 3.2. Changes in Body Weight and Food Intake After Immunization with the ssRNA Nano-Structure Adjuvant

**Figure 3:**
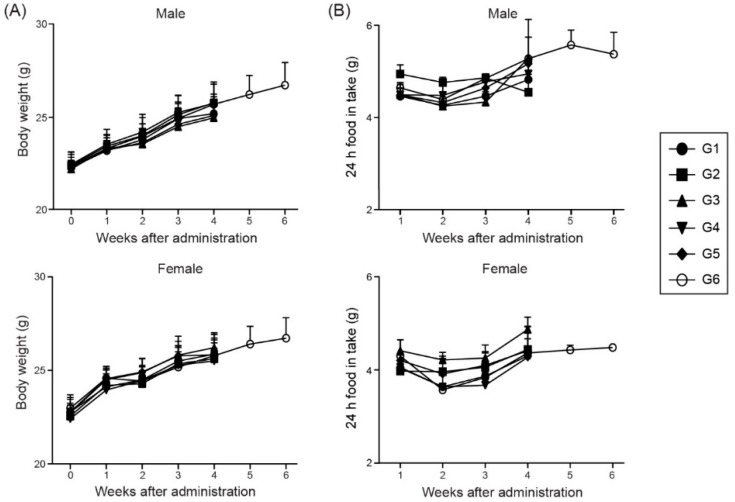
Body weights and 24 h food intake of the animals during the experiments. Bodyweight (**A**) and food intake (**B**) were measured every week following receipt of the animals. No significant changes in weights and 24-h food intake/week were found with the animals of all groups. The data shown are expressed as the mean ± SD. G1, PBS-control group; G2, 1 μg MERS S protein; G3, 200 μg ssRNA adjuvant; G4, 1 μg MERS S protein and 20 μg ssRNA adjuvant; G5, 1 μg MERS S protein and 200 μg ssRNA adjuvant; G6, 1 μg MERS S protein and 200 μg ssRNA (recovery group).

We designed an in vivo toxicity test, based on the protocol shown in Figure 1 (described in detail in the Materials and Methods section). After injecting the ssRNA nano-structure adjuvant and/or MERS S protein, we observed the behavior and symptoms of the treated mice. No specific problems were found in the injected male and female mice compared with healthy control (G1) group (data not shown). The external body weight (Figure 3A) and food intake (Figure 3B) were measured as critical toxic signs in experimental animals every week, following receipt of the animals. No significant changes were found in the weights and 24 h food intake of the male and female mice among all groups. Therefore, no animals administered ssRNA nano-structure adjuvant formulated with the MERS S protein (even with a high ssRNA concentration of 200 μg/mouse) showed any specific toxic symptoms.

### 3.3. Hematological and Serum Biochemical Parameters

Hematological results, including hematopoiesis- and leukopoiesis-associated parameters, are summarized in Table 1. Statistically significant changes in the hematological data (e.g., HGB, HCT, MCV, MCH, MCHC, and WBC) in the males and females were of small magnitude within acceptable ranges [49,50]. Therefore, these differences were not considered to be related to toxicity of the ssRNA nano-structure adjuvant. WBC differential showed that percentages of neutrophils were significantly decreased in G6 of males and females in comparison with those in G1. Male mice in G6 showed significant increase in the percentage of lymphocytes relative to the control value (G1). The percentages of monocytes and eosinophils were significantly increased in G5 of males, but these returned to normal levels in G6. No significant treatment-related changes were found with the other parameters tested in the males and females of groups treated with the ssRNA nano-structure adjuvant.

The serum biochemistry results are summarized in Table 2. Serum biochemical analysis revealed that AST levels were significantly decreased of males in G6 compared to those in G1. BUN levels were significantly decreased in G6 of males but increased in G3 and G5 of females in comparison with those in G1. Creatinine levels in G6 were lower compared to those in G1 in both sexes. Small but significant changes in TP, albumin, A/G ratio, glucose, and LDL were observed only in one sex. HDL levels were high in male mice and low in female mice of group G3, which were considered to reflect transient changes and not a concentration-dependent phenomenon. TG levels were significantly changed in both males and females compared to those of respective control groups.

### 3.4. Organ Weights and Histopathological Analysis

The data for absolute and relative organ weights are summarized in Table 3 and Table 4. Absolute liver weights of males in G6 and females in G3 and G6 were significantly higher than those of their respective control groups. Relative liver weights were significantly decreased in males of G4 but increased in females of G2 and G6 in comparison with those in the respective control groups. Absolute and relative spleen weights of males in G5 and females in G3 were significantly higher compared to those of G1. Absolute lung weights in females of G3 and G6 were higher compared to those in G1. Furthermore, relative lung weights were significantly decreased in males of G6 but increased in females of G5 in comparison with those in G1. Meanwhile, kidney weights in males of G6 and females of G2, G3, and G6 were higher than those of G1. Additionally, small and sporadic weight changes in brain, heart, and testis were found in males, and no significant weight changes in these organs were found in females. Interestingly, absolute and relative thymus weights of males in G3, G4, G5, and G6 were significantly lower compared to those of respective control groups, whereas no significant differences were found in the thymus weight among groups in females. Although these findings were regarded as toxicologically irrelevant because of the lack of remarkable histopathological correlation, further study is necessary to confirm them and to understand the mechanisms underlying the sex-related differences in thymus weight.

**Figure 4:**
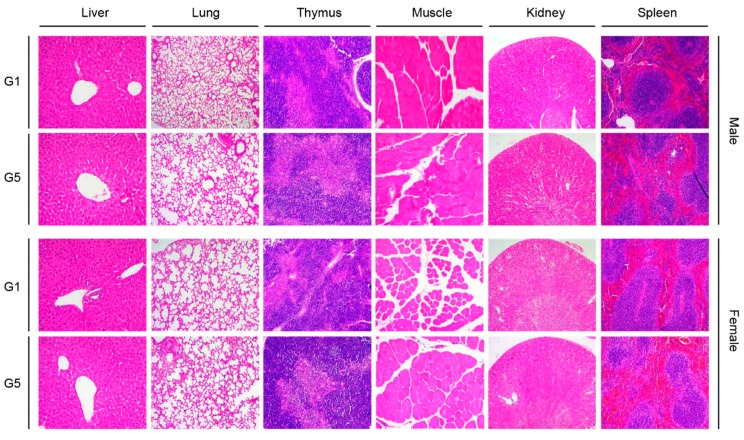
Hematoxylin and eosin (H + E) staining of the major organs of mice in the control (G1) and high-dose group (G5). The organs were stained with H + E to observe local and systemic toxicity associated with the ssRNA nano-structure adjuvant. The pictures show almost the same morphology among control (G1) and high-dose group (G5) in both male and female. G1, PBS-control group; G5, 1 μg MERS S protein and 200 μg ssRNA adjuvant.

Not only the liver, which is a typical organ studied to assess toxicity, but also the lungs, kidneys, injection sites, and immune-related organs (spleen and thymus) were stained with H + E to observe local and systemic toxicity, including cellular damage and inflammatory cell infiltration, associated with the ssRNA nano-structure adjuvant (Figure 4). In both males and females, no significant dose-related damages in the liver and lung were apparent in the high-dose group (G5, 1 μg MERS S protein and 200 μg ssRNA adjuvant) compared with G1. Renal involvement is relatively common in autoimmune diseases [51]. In this study, we did not observe any histological changes involved in the kidney damage in both sexes. The intramuscular-injection sites were also regular in appearance. Importantly, the thymus and spleen (which serve as immunological organs) did not show any treatment-related toxicity.

### 3.5. Autoimmune Disease-Related Data

**Figure 5:**
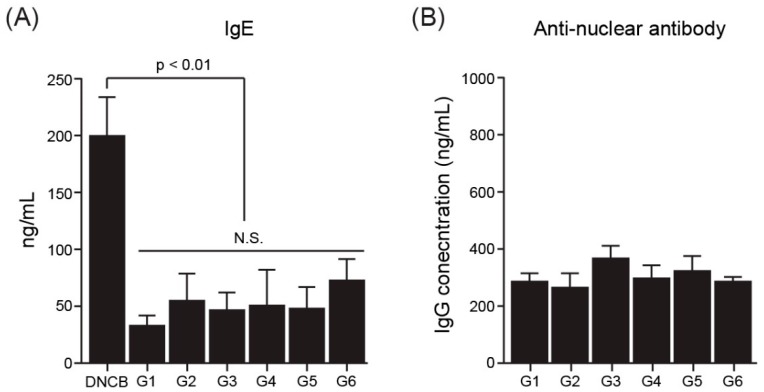
Markers of autoimmunity. (**A**) Serum IgE concentrations in immunized mice. DNCB was used as a positive control group for IgE antibody induction. (**B**) Concentrations of anti-nuclear antibodies in immunized mice, as determined by ELISA (*p < 0.01 between DNCB group and G1–G6; N.S., not significant). The ssRNA nano-structure adjuvant did not induce any significant changes in serum levels of IgE anti-nuclear antibody. G1, PBS-control group; G2, 1 μg MERS S protein; G3, 200 μg ssRNA adjuvant; G4, 1 μg MERS S protein and 20 μg ssRNA adjuvant; G5, 1 μg MERS S protein and 200 μg ssRNA adjuvant; G6, 1 μg MERS S protein and 200 μg ssRNA (recovery group).

To identify the involvement of the ssRNA nano-structure adjuvant in autoimmunity, serum levels of IgE [52] and anti-nuclear antibodies [53,54] were measured in mice after immunization with the ssRNA nano-structure adjuvant. Serum IgE antibodies in mice were examined using a mouse IgE ELISA kit (Figure 5A). As a positive control group, DNCB was used to induce allergy in mice [45]. As a result, robust IgE antibody production was not detected in any group, except for the DNCB group. The serum concentrations of anti-nuclear antibodies in treated mice were also examined using an Anti-Nuclear Antibody IgG ELISA Kit. No significant inductions in anti-nuclear antibody production were observed in groups (G3–G6) treated with the ssRNA nano-structure adjuvant compared with the PBS-treated control group (G1; Figure 5B).

### 3.6. Analysis of Inflammatory Cytokines in the Serum

**Figure 6:**
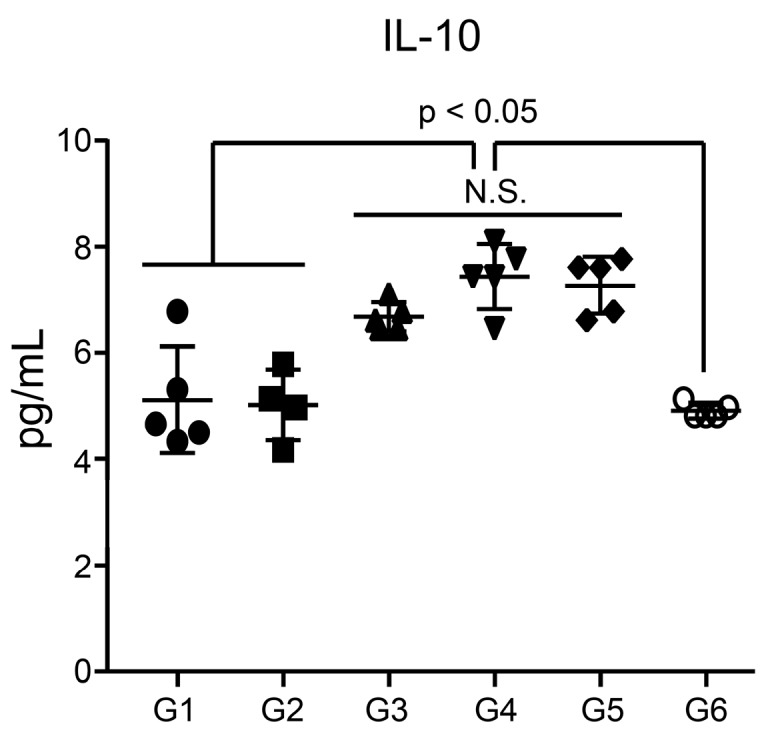
Serum levels of IL-10. Sera from mice in groups 1–5 were collected 1 day after the last immunization, and sera from mice in group 6 were collected 2 weeks after the last immunization. Cytokine levels were measured using the Magnetic Luminex^®^ Screening Assay Kit (*p < 0.05, relative to G1; between G3–G5 and G6; N.S., not significant). G1, PBS-control group; G2, 1 μg MERS S protein; G3, 200 μg ssRNA adjuvant; G4, 1 μg MERS S protein and 20 μg ssRNA adjuvant; G5, 1 μg MERS S protein and 200 μg ssRNA adjuvant; G6, 1 μg MERS S protein and 200 μg ssRNA (recovery group).

We further measured the levels of inflammation-associated cytokines, such as MCP-1, TNF-α, IL-1β, IL-6, IL12p70, and IL-10 [55]. No significant differences were found in the levels of the pro-inflammatory cytokines MCP-1, TNF-α, IL-1β, IL-6, and IL12p70 in any groups examined in this study (Figure S4A–E). Interestingly, production of the anti-inflammatory cytokine IL-10 [56] was slightly higher in the ssRNA-treated group (G3–G5) than in the other groups (Figure 6). However, the IL-10 levels in the recovery group (G6), which were sacrificed two weeks later, had decreased to comparable levels found in G1.

### 3.7. Immune Cell Activation in Splenocytes

**Figure 7:**
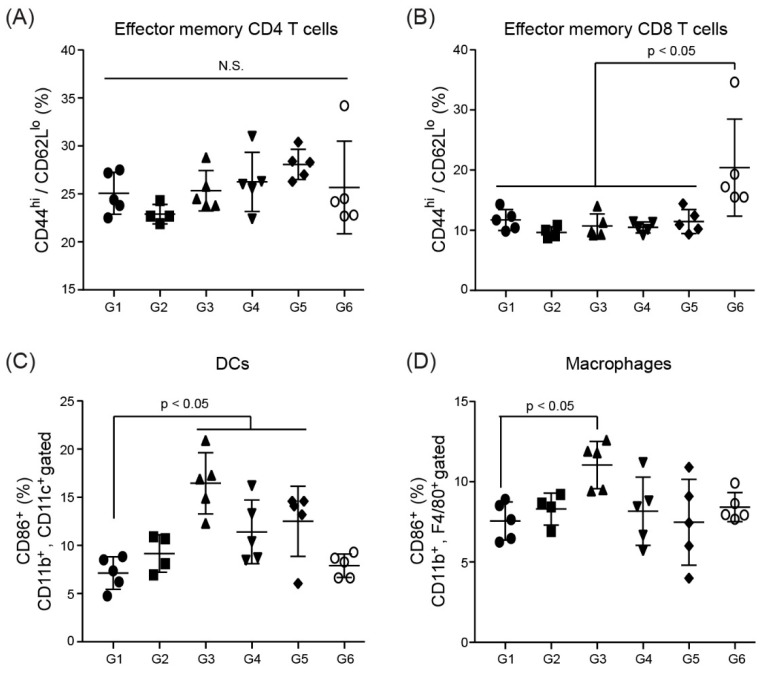
Analysis of immune cell activation after immunization with the ssRNA nano-structure adjuvant and vaccine protein. (**A**,**B**) effector memory T cells (CD4^+^ or CD8^+^, CD44^hi^, CD62L^lo^), (**C**) activated DCs (CD11b^+^, CD11c^+^, CD86^+^), and (**D**) macrophages (CD11b^+^, F4/80^+^, CD86^+^) in the spleen were counted by flow cytometry (*p < 0.05, relative to G1; N.S., not significant). G1, PBS-control group; G2, 1 μg MERS S protein; G3, 200 μg ssRNA adjuvant; G4, 1 μg MERS S protein and 20 μg ssRNA adjuvant; G5, 1 μg MERS S protein and 200 μg ssRNA adjuvant; G6, 1 μg MERS S protein and 200 μg ssRNA (recovery group).

To assess immune cell activation by the ssRNA nano-structure adjuvant, flow cytometric analysis was performed using splenocytes from immunized mice. CD44^hi^/CD62L^lo^ CD4 T cells and CD44^hi^/CD62L^lo^ CD8 T cells indicate effector memory T cells, which can rapidly differentiate into effector T cells after re-exposure to the antigen [57]. The splenocytes did not show any statistically significant differences in CD4 and CD8 T cells between any group (Figure 7A,B). The slightly higher CD8 T cell frequency in G6 versus the other groups suggested that the mice were primed for the next antigen exposure. In contrast, the ssRNA nano-structure adjuvant appeared to activate APCs, including DCs and macrophages (Figure 7C,D).

## 4. Discussion 

Recently, we developed an ssRNA nano-structure adjuvant using CrPV IGR IRES-derived RNA and found its function as an immune stimulator [39,40] in that, it stimulated a balanced Th1 and Th2 response with MERS S protein. Moreover, ssRNA nano-structure adjuvant showed a synergistic effect with alum to increase humoral and cellular immune responses [39]. Although alum is popularly used as an adjuvant for human clinical vaccines, it usually induces a profound Th2 response (humoral immune response) along with producing antibody, whereas it cannot stimulate Th1 response (cellular immune response) and sometimes triggers autoimmune response [4,39]. These previous results proved that CrPV IGR IRES-derived ssRNA nano-structure adjuvant could function as an ideal vaccine adjuvant to support the weakness of alum. Nonetheless, its safety has not been well investigated yet. Therefore, the objective of the present study was to perform a comprehensive investigation for the general toxicity profile and immunological-toxicity results on the ssRNA nano-structure adjuvant.

For an in vitro cytotoxicity study, we used liver- and lung-derived cell lines to test toxicity because they represent target organs and portals of entry for toxicants and because the liver is a detoxification site [58,59]. Moreover, we also used human normal cell lines to test toxicity because of the toxin resistance of tumor cell lines. As a result, the ssRNA nano-structure adjuvant did not elicit cellular toxicity in all tested cell lines, even with increasing ssRNA concentrations of up to 10-fold higher than we used to increase immune responses previously (20 μg/mouse) [39]. To overcome the limitation of the in vitro study, we confirmed the general toxicity and immunological toxicity of ssRNA nano-structure adjuvant in vivo. Our data indicate that the ssRNA nano-structure adjuvant formulated with the MERS S protein did not cause any treatment-related significant alteration in clinical condition, growth, or food intake. Since a broad range of toxic injuries beginning with indolent, often asymptomatic, features can progress to organ failure [60], we additionally performed hematological and histological tests to indicate systemic adverse effects of the test materials.

Following necropsy, the hematological analysis showed that the decrease of neutrophils and the increase of lymphocytes seen in the recovery group of both males and females seemed to be a typical immune response, reflecting an increase of B cells and T cells because these are typical vaccine immunization-derived responses [61,62,63]. In other hematological parameters, all treatment groups showed no significant changes within physiologically normal ranges. It indicates that the ssRNA nano-structure adjuvant did not affect hematopoiesis and leukopoiesis. In addition, no dose-related significant changes were observed in all serum biochemical parameters tested. No noticeable increases in ALT and AST levels, which are representative of liver toxicity [64], were found among the groups in both males and females. BUN and creatinine levels, which are related to renal function [65], showed little changes in both sexes. A significant difference in a fatty liver marker TG [66] was not considered to be related to its toxicity because this observed change was within acceptable ranges [50,67]. The changes in other markers were sporadic with no concentration-dependency, so these were not considered to reflect toxic changes due to the ssRNA nano-structure adjuvant. Importantly, organ weights and histological examinations of major organs such as liver, kidney, lung, spleen, thymus, and intramuscular-injection site did not cause any toxicologically significant changes involved in treatment of the ssRNA nano-structure adjuvant. Collectively, in contrast to poly I:C, which causes severe damage to the kidney and spleen [24], the ssRNA nano-structure adjuvant did not induce any organ injury in both males and females.

Alum, a commonly used adjuvant in the clinical vaccine field, sometimes induces high IgE levels, which are associated with macrophagic myofascitis, granuloma formation, and allergic reactions [13,68]. IgE is also highly elevated in systemic lupus erythematosus and other autoimmune diseases [52]. Also, anti-nuclear antibodies recognize the self as non-self and can bind the nucleus of one’s own cells, unlike conventional antibodies that bind foreign antigens, such as viral or bacterial antigens [69]. Most vertebrates commonly express anti-nuclear antibodies at very low levels [69,70] whereas a relatively high concentration of anti-nuclear antibodies indicates the induction of autoimmune disease [53,54]. In this study, the ssRNA nano-structure adjuvant caused no significant inductions in serum IgE and anti-nuclear antibodies, indicating that the ssRNA nano-structure adjuvant did not pose a risk for autoimmunity.

Inflammation-associated cytokines include MCP-1, TNF-α, IL-1β, IL-6, IL12p70, and IL-10 [55]. MCP-1, which is monocyte chemoattractant protein-1, recruit monocytes during infection to induce inflammation [71]. TNF-α induces inflammation and activates lymphocytes and leukocytes [72]. IL-1β is produced by monocytes and macrophages and plays an important role in inflammatory regulation [73]. IL-6 promotes the synthesis of acute-phase proteins, such as CRP, and is produced at the site of inflammation [74]. Overexpression of these pro-inflammatory cytokines may cause excessive inflammation or autoimmune diseases [75]. In this study, there were no statistically significant differences in the levels of the pro-inflammatory cytokines MCP-1, TNF-α, IL-1β, and IL-6 among groups. In particular, the levels of IL-12, which has been associated with autoimmunity [76], were not higher in the ssRNA-treated group than in the PBS-treated group (Figure S4E), supporting the possibility that the ssRNA nano-structure adjuvant did not induce autoimmunity, consistent with the data on serum levels of IgE and anti-nuclear antibodies shown in Figure 5. Interestingly, IL-10 levels of ssRNA nano-structure adjuvant groups (G3 to G5) compared with G1 as a control group and G6 as a recovery group was slightly increased (Figure 6). It may be counteracted to the induction of proinflammatory cytokines, regardless of no significant increases at 1 day after ssRNA treatment. However, we need further study to reveal the complicated relationship between pro- and anti-inflammatory cytokines after treatment of ssRNA non-structure adjuvant. Taken together, not only were no significant differences found in pro-inflammatory cytokine levels among the groups, but also the IL-10 levels returned to normal levels in the recovery group. These indicate that the ssRNA nano-structure adjuvant had no immunological toxicity.

No statistically significant differences in CD4 and CD8 T cells were found in the splenocytes between any group. In contrast, as has been observed with other adjuvants [77], the DC frequencies of the ssRNA nano-structure adjuvant-treated groups were higher than those in the control in the spleen, at least partly through the association with higher neutrophil levels in the hematological analysis in vivo toxicity study (Table 1). The increased DC activations found only in the G3–G5 compared with G1 as control group (Figure 7C) were accordance with previous data [39], which showed that ssRNA nano-structure adjuvant transiently triggered DC activation in the injected site. This may have been caused by sacrificing the mice on the first day after the last immunization because DC activation is an early event after vaccine injection [3,78]. The frequencies of DCs in the recovery group returned to levels found in the control group (Figure 7C). Since a short immune duration was observed without toxicological differences in organ weights or histopathology, it is considered that our ssRNA nano-structure adjuvant did not elicit immunological toxicity. Moreover, in draining lymph nodes, no significant difference occurred between groups in terms of both DCs and macrophages (data not shown). These data indicated that our ssRNA nano-structure adjuvant performed successfully as safe adjuvant.

## 5. Conclusions

Previously, the ssRNA nano-structure adjuvant tested in this study was shown to effectively elicit immune enhancement in mice and non-human primates, based on balanced Th1 and Th2 responses [39,40]. In this study, we showed that the adjuvant did not cause weight loss or abnormal behavior in mice after immunization, even at high doses. Moreover, it did not increase any hematological and biochemical parameters and did not change the histopathological patterns compared with the control group. In addition, the ssRNA nano-structure adjuvant did not promote sustained pro-inflammatory cytokines and excess immune cell activation for a long time, as these indicators quickly disappeared after immunization. Instead, our results appeared to reflect an effective ‘hit-and-run’ strategy for stimulating immune responses. Therefore, the ssRNA nano-structure can function as a safe adjuvant with high vaccine efficacy.

## Figures and Tables

**Table 1:** Hematological data of male and female BALB/c mice intramuscularly administered the ssRNA nano-structure adjuvant.

	Group 1	Group 2	Group 3	Group 4	Group 5	Group 6
***Male***						
RBC (×10^6^ cells/μL)	10.3 ± 0.5	10.0 ± 0.7	10.3 ± 0.6	10.2 ± 0.2	10.3 ± 0.5	10.1 ± 0.3
HGB (g/dL)	14.8 ± 0.6	14.5 ± 0.9	14.9 ± 0.9	14.6 ± 0.4	14.7 ± 0.8	14.4 ± 0.4
HCT (%)	50.6 ± 2.5	48.7 ± 3.2	51.4 ± 3.2	50.5 ± 1.6	51.4 ± 3.1	48.5 ± 1.7
MCV (fL)	49.1 ± 0.5	48.8 ± 0.3	49.8 ± 0.2 *	49.7 ± 0.7	49.9 ± 0.4 *	48.2 ± 0.5 *
MCH (pg)	14.4 ± 0.1	14.6 ± 0.1 *	14.4 ± 0.1	14.4 ± 0.1	14.2 ± 0.1	14.3 ± 0.1
MCHC (g/dL)	29.3 ± 0.3	29.8 ± 0.2 *	29.0 ± 0.3	28.9 ± 0.2	28.6 ± 0.3 *	29.8 ± 0.2 *
PLT (×10^3^ cells/μL)	923.0 ± 61.0	929.0 ± 54.0	1128.0 ± 206.0	970.0 ± 20.0	956.0 ± 76.0	899.0 ± 43.0
WBC (×10^3^ cells/μL)	3.2 ± 1.3	3.8 ± 1.2	2.2 ± 0.7	2.4 ± 0.7	2.1 ± 0.6	3.3 ± 0.7
Neutrophils (%)	27.7 ± 7.1	26.9 ± 2.2	40.5 ± 4.4 *	30.6 ± 4.2	34.0 ± 8.1	18.0 ± 2.2 *
Lymphocytes (%)	69.5 ± 7.2	70.5 ± 2.7	56.9 ± 4.7 *	65.5 ± 3.8	61.3 ± 7.5	78.6 ± 1.9 *
Monocytes (%)	0.8 ± 0.3	0.7 ± 0.4	0.9 ± 0.4	0.7 ± 0.3	1.3 ± 0.3 *	0.5 ± 0.4
Eosinophils (%)	1.9 ± 0.8	1.8 ± 0.6	1.6 ± 1.1	3.0 ± 0.7	3.3 ± 1.0 *	2.9 ± 0.8
Basophils (%)	0.1 ± 0.2	0.1 ± 0.2	0.0 ± 0.0	0.2 ± 0.2	0.1 ± 0.2	0.1 ± 0.2
***Female***						
RBC (×10^6^ cells/μL)	10.3 ± 0.7	9.8 ± 0.4	9.9 ± 0.3	9.8 ± 0.3	9.7 ± 0.4	9.6 ± 0.3
HGB (g/dL)	15.4 ± 1.0	14.6 ± 0.4	14.6 ± 0.5	14.4 ± 0.3	14.3 ± 0.6	14.2 ± 0.3 *
HCT (%)	52.3 ± 4.1	50.4 ± 1.1	49.6 ± 1.7	48.3 ± 1.4	48.1 ± 2.3	47.7 ± 1.4 *
MCV (fL)	50.7 ± 1.0	51.5 ± 0.7	50.3 ± 0.4	49.3 ± 0.4 *	49.6 ± 0.8	49.5 ± 0.4
MCH (pg)	14.9 ± 0.2	14.9 ± 0.1	14.8 ± 0.1	14.7 ± 0.2	14.8 ± 0.1	14.8 ± 0.3
MCHC (g/dL)	29.4 ± 0.3	28.9 ± 0.2 *	29.4 ± 0.2	29.9 ± 0.2 *	29.8 ± 0.3	29.9 ± 0.4
PLT (×10^3^ cells/μL)	777.0 ± 84.0	742.0 ± 145.0	857.0 ± 50.0	820.0 ± 99.0	834.0 ± 42.0	780.0 ± 83.0
WBC (×10^3^ cells/μL)	2.7 ± 0.7	3.8 ± 0.5 *	1.6 ± 0.4 *	3.0 ± 0.2	1.6 ± 0.6 *	2.3 ± 0.6
Neutrophils (%)	28.5 ± 4.5	26.4 ± 3.6	27.6 ± 3.2	30.6 ± 6.4	37.8 ± 11.8	19.9 ± 6.4 *
Lymphocytes (%)	67.5 ± 4.7	68.1 ± 3.2	66.9 ± 3.4	64.5 ± 5.8	59.5 ± 10.5	75.3 ± 5.9
Monocytes (%)	0.7 ± 0.2	1.3 ± 0.3 *	1.2 ± 0.3 *	1.3 ± 0.6	1.0 ± 0.4	0.8 ± 0.2
Eosinophils (%)	3.3 ± 1.5	4.1 ± 1.7	4.1 ± 0.8	3.4 ± 0.6	1.7 ± 1.1	4.0 ± 1.2
Basophils (%)	0.0 ± 0.0	0.1 ± 0.1	0.2 ± 0.4	0.1 ± 0.2	0.0 ± 0.0	0.1 ± 0.1

The data shown are expressed as the mean ± SD. *p < 0.05, relative to Group 1. Group 1, PBS-control group; Group 2, 1 μg MERS S protein; Group 3, 200 μg ssRNA adjuvant; Group 4, 1 μg MERS S protein and 20 μg ssRNA adjuvant; Group 5, 1 μg MERS S protein and 200 μg ssRNA adjuvant; Group 6, 1 μg MERS S protein and 200 μg ssRNA (recovery group).

**Table 2:** Serum biochemical data for male and female BALB/c mice intramuscularly administered the ssRNA nano-structure adjuvant.

	Group 1	Group 2	Group 3	Group 4	Group 5	Group 6
***Male***						
ALT (U/L)	53.7 ± 52.5	36.0 ± 8.9	36.2 ± 3.1	32.7 ± 3.5	39.1 ± 5.4	25.9 ± 4.6
AST (U/L)	73.0 ± 20.3	66.3 ± 9.3	67.1 ± 12.6	66.5 ± 14.1	79.5 ± 15.3	51.5 ± 3.6 *
BUN (mg/dL)	31.7 ± 6.4	38.5 ± 6.7	27.3 ± 3.6	32.2 ± 7.1	35.3 ± 8.8	24.1 ± 1.0 *
Creatinine (mg/dL)	0.2 ± 0.0	0.2 ± 0.0	0.2 ± 0.1	0.2 ± 0.0	0.2 ± 0.0	0.1 ± 0.0 *
TP (g/dL)	5.8 ± 0.5	5.6 ± 0.3	5.5 ± 0.3	5.5 ± 0.2	5.3 ± 0.3	5.1 ± 0.5
Albumin (g/dL)	3.3 ± 0.2	3.2 ± 0.3	3.2 ± 0.1	3.3 ± 0.1	3.0 ± 0.1 *	3.2 ± 0.2
A/G	1.4 ± 0.2	1.3 ± 0.2	1.4 ± 0.1	1.5 ± 0.1	1.4 ± 0.1	1.7 ± 0.2 *
T-Chol (mg/dL)	148.0 ± 9.0	144.0 ± 18.0	154.0 ± 6.0	148.0 ± 8.0	152.0 ± 16.0	146.0 ± 12.0
TG (mg/dL)	99.0 ± 14.0	121.0 ± 33.0	125.0 ± 24.0	84.0 ± 10.0	49.0 ± 21.0 *	150.0 ± 38.0 *
Glucose (mg/dL)	165.7 ± 21.7	200.0 ± 35.5	178.2 ± 20.5	174.5 ± 22.9	122.8 ± 40.3	169.2 ± 20.3
HDL (mg/dL)	113.3 ± 6.2	117.1 ± 10.8	129.1 ± 7.0 *	117.3 ± 5.5	124.2 ± 13.5	106.6 ± 8.2
LDL (mg/dL)	5.2 ± 1.3	5.5 ± 1.0	6.2 ± 0.9	4.7 ± 0.7	6.2 ± 1.1	6.2 ± 0.9
***Female***						
ALT (U/L)	32.6 ± 3.2	35.2 ± 5.3	29.1 ± 2.6	35.5 ± 7.5	29.8 ± 2.9	35.8 ± 8.5
AST (U/L)	69.3 ± 6.0	76.3 ± 5.9	63.9 ± 3.8	75.8 ± 11.6	63.9 ± 6.4	74.3 ± 9.6
BUN (mg/dL)	26.3 ± 3.4	32.6 ± 11.6	44.1 ± 6.0 *	28.8 ± 5.6	35.1 ± 4.7 *	28.0 ± 4.1
Creatinine (mg/dL)	0.2 ± 0.1	0.2 ± 0.0	0.2 ± 0.0	0.2 ± 0.0	0.2 ± 0.0	0.1 ± 0.0 *
TP (g/dL)	5.9 ± 0.6	6.0 ± 0.5	5.0 ± 0.4 *	5.6 ± 0.2	5.0 ± 0.4 *	5.0 ± 0.3 *
Albumin (g/dL)	3.3 ± 0.3	3.3 ± 0.2	3.1 ± 0.1	3.0 ± 0.6	3.1 ± 0.1	3.2 ± 0.2
A/G	1.3 ± 0.4	1.3 ± 0.3	1.7 ± 0.3	1.2 ± 0.4	1.8 ± 0.5	1.8 ± 0.3
T-Chol (mg/dL)	109.0 ± 17.0	105.0 ± 13.0	110.0 ± 7.0	101.0 ± 23.0	109.0 ± 9.0	105.0 ± 13.0
TG (mg/dL)	148.0 ± 18.0	109.0 ± 37.0	148.0 ± 36.0	100.0 ± 22.0 *	112.0 ± 7.0 *	130.0 ± 49.0
Glucose (mg/dL)	165.2 ± 21.0	176.5 ± 50.5	190.0 ± 37.0	196.6 ± 29.4	197.7 ± 31.5	216.9 ± 9.3 *
HDL (mg/dL)	92.9 ± 4.2	92.2 ± 4.1	82.4 ± 5.0 *	91.1 ± 5.5	83.9 ± 11.5	80.8 ± 10.8 *
LDL (mg/dL)	9.2 ± 0.6	9.7 ± 0.8	9.6 ± 1.4	9.7 ± 0.6	9.3 ± 1.4	8.1 ± 0.8 *

The data shown are expressed as the mean ± SD. *p < 0.05, relative to Group 1. Group 1, PBS-control group; Group 2, 1 μg MERS S protein; Group 3, 200 μg ssRNA adjuvant; Group 4, 1 μg MERS S protein and 20 μg ssRNA adjuvant; Group 5, 1 μg MERS S protein and 200 μg ssRNA adjuvant; Group 6, 1 μg MERS S protein and 200 μg ssRNA (recovery group).

**Table 3:** Organ weights of male BALB/C mice intramuscularly administered the ssRNA nano-structure adjuvant.

		Group 1	Group 2	Group 3	Group 4	Group 5	Group 6
***Male***							
Liver	(g)	1.06 ± 0.11	1.08 ± 0.07	0.99 ± 0.07	0.97 ± 0.04	1.02 ± 0.03	1.25 ± 0.09 *
	(%BW)	4.54 ± 0.45	4.31 ± 0.25	4.14 ± 0.22	4.04 ± 0.05 *	4.26 ± 0.09	4.62 ± 0.16
Spleen	(g)	0.09 ± 0.01	0.10 ± 0.01	0.09 ± 0.01	0.09 ± 0.01	0.10 ± 0.01 *	0.09 ± 0.01
	(%BW)	0.38 ± 0.03	0.39 ± 0.03	0.39 ± 0.05	0.39 ± 0.05	0.44 ± 0.03 *	0.35 ± 0.02
Lung	(g)	0.16 ± 0.01	0.16 ± 0.02	0.17 ± 0.01	0.15 ± 0.01	0.15 ± 0.02	0.15 ± 0.01
	(%BW)	0.67 ± 0.07	0.63 ± 0.07	0.70 ± 0.03	0.63 ± 0.05	0.63 ± 0.08	0.55 ± 0.04 *
Kidney(L)	(g)	0.18 ± 0.02	0.19 ± 0.01	0.19 ± 0.02	0.18 ± 0.01	0.17 ± 0.01	0.21 ± 0.01 *
	(%BW)	0.79 ± 0.08	0.76 ± 0.02	0.79 ± 0.06	0.75 ± 0.03	0.71 ± 0.03	0.76 ± 0.03
Kidney(R)	(g)	0.19 ± 0.02	0.19 ± 0.01	0.19 ± 0.01	0.18 ± 0.01	0.17 ± 0.01	0.21 ± 0.01 *
	(%BW)	0.80 ± 0.09	0.77 ± 0.02	0.81 ± 0.04	0.74 ± 0.03	0.73 ± 0.03	0.77 ± 0.04
Thymus	(g)	0.06 ± 0.01	0.05 ± 0.01	0.04 ± 0.01 *	0.04 ± 0.01 *	0.03 ± 0.01 *	0.04 ± 0.01 *
	(%BW)	0.24 ± 0.05	0.19 ± 0.03	0.17 ± 0.04 *	0.18 ± 0.01 *	0.14 ± 0.01 *	0.15 ± 0.02 *
Brain	(g)	0.43 ± 0.02	0.43 ± 0.02	0.42 ± 0.01	0.43 ± 0.01	0.42 ± 0.03	0.44 ± 0.02
	(%BW)	1.83 ± 0.08	1.70 ± 0.09 *	1.74 ± 0.06	1.78 ± 0.02	1.77 ± 0.13	1.62 ± 0.04 *
Heart	(g)	0.13 ± 0.01	0.15 ± 0.01 *	0.14 ± 0.01	0.13 ± 0.01	0.13 ± 0.01	0.15 ± 0.01 *
	(%BW)	0.58 ± 0.04	0.60 ± 0.04	0.57 ± 0.03	0.54 ± 0.03	0.56 ± 0.02	0.54 ± 0.03
Testis(L)	(g)	0.10 ± 0.01	0.09 ± 0.01	0.10 ± 0.01	0.10 ± 0.01	0.10 ± 0.01	0.09 ± 0.04
	(%BW)	0.44 ± 0.04	0.37 ± 0.04 *	0.42 ± 0.04	0.41 ± 0.03	0.43 ± 0.02	0.33 ± 0.14
Testis(R)	(g)	0.10 ± 0.01	0.11 ± 0.02	0.10 ± 0.01	0.10 ± 0.01	0.10 ± 0.01	0.09 ± 0.04
	(%BW)	0.44 ± 0.05	0.42 ± 0.05	0.44 ± 0.04	0.42 ± 0.02	0.42 ± 0.02	0.33 ± 0.14

The data shown are expressed as the mean ± SD. *p < 0.05, relative to Group 1. Group 1, PBS-control group; Group 2, 1 μg MERS S protein; Group 3, 200 μg ssRNA adjuvant; Group 4, 1 μg MERS S protein and 20 μg ssRNA adjuvant; Group 5, 1 μg MERS S protein and 200 μg ssRNA adjuvant; Group 6, 1 μg MERS S protein and 200 μg ssRNA (recovery group).

**Table 4:** Organ weights of female BALB/C mice intramuscularly administered the ssRNA nano-structure adjuvant.

		Group 1	Group 2	Group 3	Group 4	Group 5	Group 6
***Female***							
Liver	(g)	0.78 ± 0.07	0.91 ± 0.11	0.89 ± 0.07 *	0.77 ± 0.04	0.74 ± 0.09	0.94 ± 0.05 *
	(%BW)	3.92 ± 0.23	4.39 ± 0.33 *	4.17 ± 0.19	3.80 ± 0.13	3.80 ± 0.23	4.40 ± 0.09 *
Spleen	(g)	0.10 ± 0.01	0.10 ± 0.01	0.12 ± 0.01 *	0.10 ± 0.01	0.10 ± 0.01	0.10 ± 0.01
	(%BW)	0.48 ± 0.04	0.49 ± 0.03	0.55 ± 0.04 *	0.49 ± 0.02	0.50 ± 0.04	0.46 ± 0.04
Lung	(g)	0.13 ± 0.01	0.14 ± 0.01	0.15 ± 0.01 *	0.14 ± 0.01	0.14 ± 0.01	0.14 ± 0.01 *
	(%BW)	0.64 ± 0.03	0.66 ± 0.03	0.68 ± 0.03	0.69 ± 0.06	0.74 ± 0.03 *	0.67 ± 0.03
Kidney(L)	(g)	0.12 ± 0.01	0.13 ± 0.01 *	0.13 ± 0.01	0.12 ± 0.01	0.12 ± 0.01	0.13 ± 0.01 *
	(%BW)	0.59 ± 0.03	0.63 ± 0.03	0.62 ± 0.03	0.60 ± 0.02	0.64 ± 0.05	0.61 ± 0.02
Kidney(R)	(g)	0.12 ± 0.01	0.14 ± 0.01 *	0.14 ± 0.01 *	0.12 ± 0.01	0.12 ± 0.01	0.13 ± 0.01 *
	(%BW)	0.61 ± 0.02	0.65 ± 0.04	0.65 ± 0.02 *	0.60 ± 0.04	0.63 ± 0.03	0.63 ± 0.04
Thymus	(g)	0.04 ± 0.01	0.03 ± 0.01	0.04 ± 0.01	0.04 ± 0.01	0.03 ± 0.01	0.04 ± 0.01
	(%BW)	0.20 ± 0.02	0.16 ± 0.04	0.20 ± 0.02	0.20 ± 0.02	0.18 ± 0.02	0.19 ± 0.02
Brain	(g)	0.44 ± 0.02	0.44 ± 0.02	0.45 ± 0.01	0.44 ± 0.01	0.43 ± 0.01	0.44 ± 0.02
	(%BW)	2.18 ± 0.07	2.12 ± 0.10	2.11 ± 0.07	2.17 ± 0.02	2.25 ± 0.18	2.06 ± 0.16
Heart	(g)	0.11 ± 0.01	0.11 ± 0.01	0.09 ± 0.05	0.11 ± 0.01	0.11 ± 0.01	0.11 ± 0.01
	(%BW)	0.53 ± 0.02	0.55 ± 0.02	0.44 ± 0.21	0.53 ± 0.03	0.58 ± 0.07	0.52 ± 0.02

The data shown are expressed as the mean ± SD. *p < 0.05, relative to Group 1. Group 1, PBS-control group; Group 2, 1 μg MERS S protein; Group 3, 200 μg ssRNA adjuvant; Group 4, 1 μg MERS S protein and 20 μg ssRNA adjuvant; Group 5, 1 μg MERS S protein and 200 μg ssRNA adjuvant; Group 6, 1 μg MERS S protein and 200 μg ssRNA (recovery group).

## Supplementary Materials

The following are available online at https://www.mdpi.com/1999-4923/11/9/464/s1, Figure S1: Brief structure of CrPV IGR IRES-derived ssRNA nano-structure adjuvant. Figure S2: Dose-dependent cell viabilities of various cell lines treated with the ssRNA nano-structure adjuvant, using MTT assays. Relative viabilities of (A) A549 cells and (B) HepG2 cells were compared to negative control (0 concentration of ssRNA nano-structure adjuvant) from 24 h to 72 h, based on the ssRNA concentration. Figure S3: Pro-inflammatory cytokine induction in RAW 264.7 cells after treatment with poly I:C and ssRNA nano-structure adjuvant. Supplementary method: Real-time PCR for proinflammatory cytokine in RAW 264.7 cells after treatment with poly I:C and ssRNA nano-structure adjuvant. Figure S4: Serum levels of MCP-1, TNF-α, IL-1β, IL-6, and IL12p70. Sera from mice in groups 1–5 were collected 1 day after the last immunization, and sera from mice in group 6 were collected 2 weeks after the last immunization.
