# Supplementary material for: Comprehensive Analysis of the Safety Profile of a Single-Stranded RNA Nano-Structure Adjuvant
Source: Pharmaceutics. 2019 Sep 7;11(9):464. doi: 10.3390/pharmaceutics11090464 (PMC6781302; doi:10.3390/pharmaceutics11090464)
Supplement: Supplementary file 1 [file pharmaceutics-11-00464-s001.pdf]

pharmaceutics

Supplementary Materials: Comprehensive Analysis of the Safety Profile of a Single-Stranded RNA Nano-Structure Adjuvant

Hyeong-Jun Park, Hae Li Ko, Dong-Hoon Won, Da-Bin Hwang, Yoo-Sub Shin, Hye-Won Kwak, Hye-Jung Kim, Jun-Won Yun and Jae-Hwan Nam

1. Supplementary Method

1.1. Cell Culture and CELL-Viability Assay of A549 and HepG2 Cell Line

HepG2 cells, derived from a human hepatocellular carcinoma, and A549 cells, derived from human lung carcinoma, were obtained from Korean Cell Line Bank (Seoul, Korea). HepG2 and A549 cells were cultured in Dulbecco's modified Eagle medium (HyClone™) with 10% fetal bovine serum (FBS, Corning, NY, USA) and 1% antibiotic-antimycotic (Anti-anti, Gibco, CA, USA). All cells were cultured in a humidified 37 °C incubator with 5% CO_2.

In vitro cell-viability testing was performed with a 3-(4,5-demerthylthiazol-2-yl)-2,5-diphenyltetrazolium bromide (MTT) assay. A549 and HepG2 cells were seeded in 24-well plates at densities of 2×10^5 cells/well or 3×10^5 cells/well. After 24 h, the medium without 10% fetal bovine serum was replaced and then treated with 10, 20, 100, or 200 µg of the ssRNA nano-structure adjuvant per well. Cell viabilities were determined at different time points (24, 48, and 72 h) by performing MTT assays. The viability of cells in each well was measured in terms of the optical density at a wavelength of 570 nm, using a GloMax® microplate reader (Promega, WI, USA).

1.2. Real Time PCR for Proinflammatory Cytokine in RAW 264.7 Cells After Treatment with Poly I:C and ssRNA Nano-Structure Adjuvant

RAW 264.7 cells were seeded in 6-well plates at 5×10^5 cells/well. After 24 h, the media was removed and 20 µg of the ssRNA nano-structure adjuvant or poly I:C as a positive control was added to each well. After 3 h, RNA was extracted from treated RAW 264.7 cells using Tri-RNA reagent (FAVORGEN®, Kaohsiung, Taiwan) and was used as the template for cDNA synthesis using a ReverTra Ace® qPCR RT Master Mix (TOYOBO, Osaka, Japan). Quantitative RT-PCR analysis was performed using an iCycler MyiQ Single Color Real-Time PCR Detection System instrument (Bio-Rad, Hercules, CA, USA) with SYBR Premix Ex Taq II (TAKARA, Shiga, Japan) normalized with GAPDH mRNA. The real-time qPCR primers used were: *Mouse GAPDH–Forward* 5′-CGTCCCGTAGACAAAATGGT-3′, *Mouse GAPDH–Reverse* 5′-TCAATGAAGGGGTCGTTGAT-3′; *Mouse TNF-α–Forward* 5′-ATGAGCACAGAAAGCATGAT-3′, *Mouse TNF-α–Reverse* 5′-TACAGGCTTGTCACTCGAAT-3′, *Mouse IL-1β – Forward* 5′-ATGGCAACTGTTCCTGAACT-3′, *Mouse IL-1β–Reverse* 5′-CAGGACAGGTATAGATTCTT-3′; *Mouse IL-6–Forward* 5′-TTCCATCCAGTTGCCTTCTT-3′, *Mouse IL-6–Reverse* 5′-ATTTCCACGATTTCCCAGAG-3′.

1.3. Analysis of Pro-Inflammatory Cytokines

Serum samples were prepared by centrifugation (2000× *g*, 30 min) from the last collected blood sample of each group and stored at −80 °C. The concentrations of IL-1β, IL-6, IL-12 p70, TNF-α, and MCP-1 were analyzed in each sample, using the Magnetic Luminex® Screening Assay Kit (R&D Systems, Inc., MN, USA) in accordance with the manufacturer's instructions.

2. Supplementary Figures

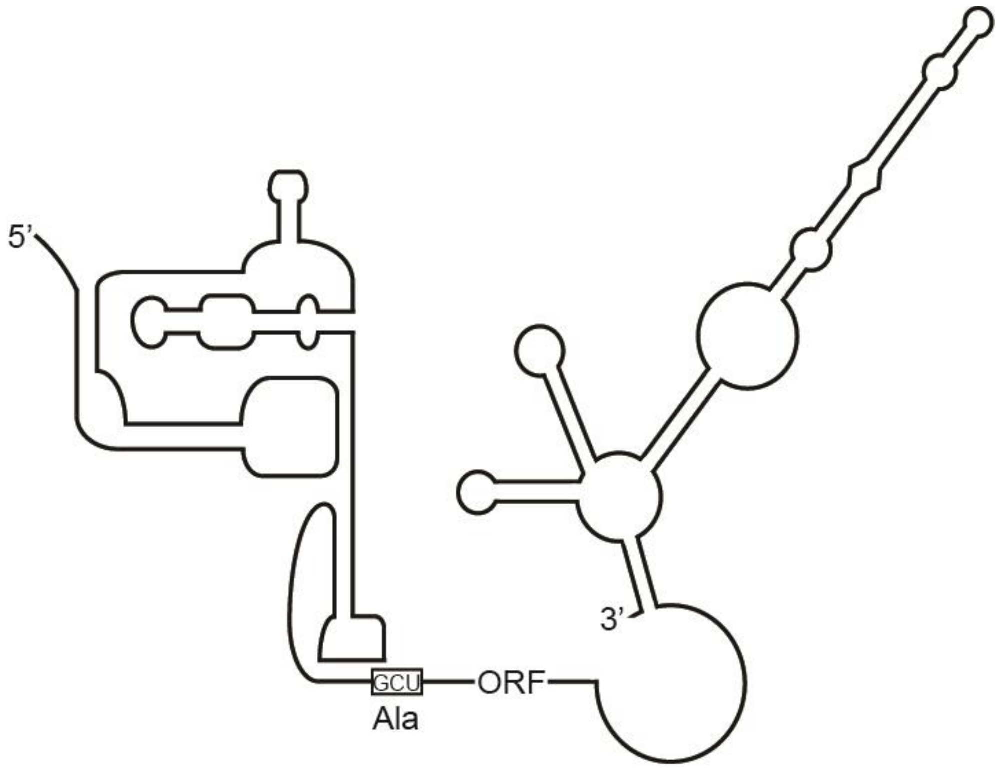

Figure S1. Brief structure of CrPV IGR IRES-derived ssRNA nano-structure adjuvant.

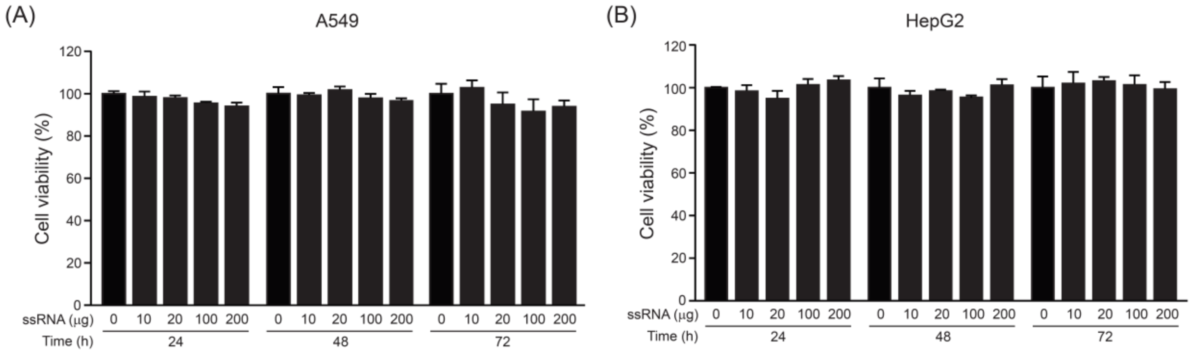

Figure S2. Dose-dependent cell viabilities of various cell lines treated with the ssRNA nano-structure adjuvant, using MTT assays. Relative viabilities of (**A**) A549 cells and (**B**) HepG2 cells were compared to negative control (0 concentration of ssRNA nano-structure adjuvant) from 24 h to 72 h, based on the ssRNA concentration.

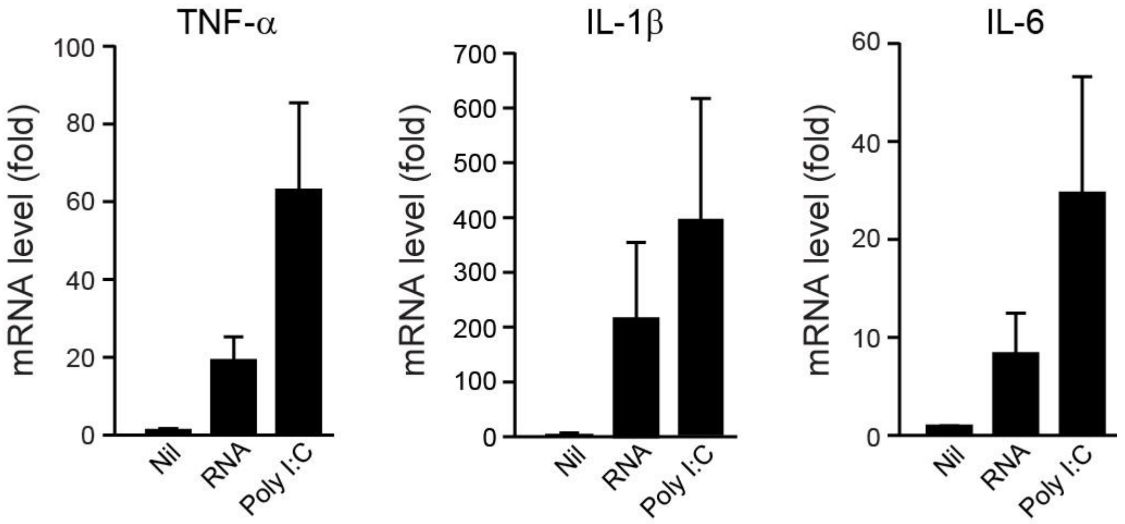

Figure S3. Pro-inflammatory cytokine induction in RAW 264.7 cells after treatment with poly I:C and ssRNA nano-structure adjuvant. Supplementary method: Real-time PCR for proinflammatory cytokine in RAW 264.7 cells after treatment with poly I:C and ssRNA nano-structure adjuvant.

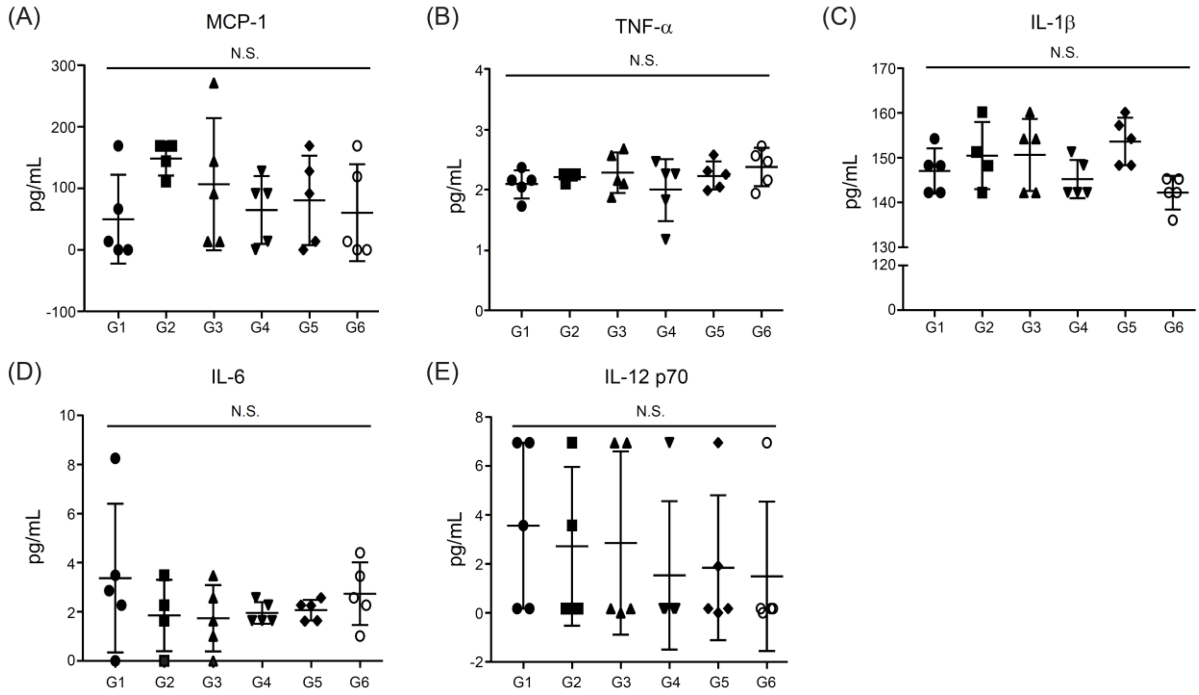

Figure S4. Serum levels of MCP-1, TNF-α, IL-1β, IL-6, and IL12p70. Sera from mice in groups 1–5 were collected 1 day after the last immunization, and sera from mice in group 6 were collected 2 weeks after the last immunization.
